# Effect of Humic Acid on As Redox Transformation and Kinetic Adsorption onto Iron Oxide Based Adsorbent (IBA)

**DOI:** 10.3390/ijerph111010710

**Published:** 2014-10-16

**Authors:** Hoda Fakour, Tsair-Fuh Lin

**Affiliations:** Department of Environmental Engineering, National Cheng Kung University, Tainan 701, Taiwan; E-Mail: fakour.h@gmail.com

**Keywords:** As, natural organic matter, adsorption rate, speciation, ternary system, iron oxide based adsorbent

## Abstract

Due to the importance of adsorption kinetics and redox transformation of arsenic (As) during the adsorption process, the present study elucidated natural organic matter (NOM) effects on As adsorption-desorption kinetics and speciation transformation. The experimental procedures were conducted by examining interactions of arsenate and arsenite with different concentrations of humic acid (HA) as a model representative of NOM, in the presence of iron oxide based adsorbent (IBA), as a model solid surface in three environmentally relevant conditions, including the simultaneous adsorption of both As and HA onto IBA, HA adsorption onto As-presorbed IBA, and As adsorption onto HA-presorbed IBA. Experimental adsorption-desorption data were all fitted by original and modified Lagergren pseudo-first and -second order adsorption kinetic models, respectively. Weber’s intraparticle diffusion was also used to gain insight into the mechanisms and rate controlling steps, which the results suggested that intraparticle diffusion of As species onto IBA is the main rate-controlling step. Different concentrations of HA mediated the redox transformation of As species, with a higher oxidation ability than reduction. The overall results indicated the significant effect of organic matter on the adsorption kinetics and redox transformation of As species, and consequently, the fate, transport and mobility of As in different environmentally relevant conditions.

## 1. Introduction

Arsenic (As) is widely recognized as a toxic carcinogen and a threat to the world’s water resources [1,2]. Exposure through drinking water, food or air causes different adverse health effects, which depend not only on the level of exposure but also on the As speciation [3,4]. Naturally occurring As is composed of inorganic and organic forms, where the former being more toxic than the latter, with arsenite (As(III)) reported to be more mobile and toxic than arsenate (As(V)) [5]. The fate and bioavailability of As and many other heavy metals in contaminated environments are mainly controlled by adsorption reactions on the soil minerals’ surfaces [6]. Many adsorbent media in aquatic environments are known to have a strong reaction with dissolved substances affecting the transfer of many pollutants, including As [7]. Iron oxides are relatively abundant in natural systems, such as soils, rocks and ground water [8] and are known to have a strong affinity for As in water due to their high surface areas and partially positive charges [9]. Different solids coated with iron oxides have been used in adsorption processes, including zeolite [10], montmorillonite [11], cement [12], activated carbon [13], and sand [14]. Adsorption and desorption reactions between As and iron-oxide surfaces are thus particularly important due to the widespread presence of iron oxides in the hydro-geologic environment as coatings on other solids result in a considerable adsorption of As to iron-oxide surfaces [15,16].

Most technologies for As removal from contaminated water are based on the oxidation of the As(III) to the less toxic and mobile As(V) (e.g., [17,18,19]). Research effort has been thus focused on the oxidation of arsenite to arsenate. It is equally noteworthy to investigate environmental parameters that affect the redox state of As species [20]. A better understanding of the redox cycling of As species along with effective factors could result in the development of more eco-friendly and efficient As removal technologies, particularly for impoverished communities severely threatened by As contamination.

Applications of iron oxide based adsorbents in As removal have been well documented [21,22,23]. However, impact of organic matter coming from soil and groundwater on the adsorption of As is studied to a much lesser extent whereas its presence may influence arsenic speciation and biogeochemistry [24,25]. Natural organic matter (NOM) is an intrinsic complex mixture of single and polyfunctional organic acids derived from the decomposition of terrestrial and aquatic once-living organisms [26]. Widespread in aquatic environments, NOM is highly reactive towards both metals and (hydro)oxide surfaces, playing an important role in controlling fate, speciation, bioavailability and transport of both organic and inorganic pollutants [27,28]. NOM includes both humic and non-humic fractions. The humic fraction is composed of humic acid (HA), fulvic acid (FA) and humin, collectively known as humic substances (HS), which constitute a major fraction of the NOM (60%–90% of dissolved organic carbon) in aquatic systems [29,30,31,32,33]. HA comprises about 70% of NOM [34,35,36,37] and has been thus studied by many researchers as a model compound for natural organic matter in water [38,39,40,41,42]. HA is a kind of chemically reactive large molecular weight humic substance (ranging from 2 kDa up to over 500 kDa) [38,43,44,45,46] and since HA affects metal speciation, it may influence the fate and transport of As species as well [47]. Numerous studies have already been conducted on As adsorption onto oxy-hydroxides and on the effect of NOM on its adsorption (e.g., [16,48,49,50]). For example, Redman *et al.* [47] investigated the effect of different-originated NOM on the As speciation and sorption onto hematite and showed the significant influence of organic matter on the attainment of sorption equilibrium as well as redox transformation of As species. They observed the reduction of As(V) to free As (III) by the Inangahua River NOM, and oxidation of As(III) to free As(V) by all other experimental NOM samples. As they reported, NOM structures themselves act as a redox-active agents influencing the arsenic speciation, through quinone or other functional groups that had been previously oxidized or reduced in their original environments. In addition, their findings also suggest that hematite may act as a surface catalyst or as an electron-transfer intermediate in the redox process. Martin *et al.* [51] reported that the formation of the humate ferrihydrite-kaolinite complex system influenced the reactivity of ferrihydrite toward arsenite and arsenate, resulting in a lower adsorption maximum as well as a decrease in the affinity of the adsorbent surfaces toward both As species. Although the interaction of As and organic matter has been sparsely reported in the literature (e.g., [25,52,53]), the primary focus has been on either a single system containing only As and NOM or competition between NOM and As for sorption sites on natural and/or synthetic minerals and often the redox transformations have not been accounted for. On the other hand, most studies regarding adsorption, desorption and competition between As and natural organic matter on solid surfaces have focused on experimental data obtained under equilibrium or near-equilibrium conditions with less information on the kinetics of the undergoing processes. This kind of information is applicable and valuable for a better understanding of the fate and mechanisms of the adsorption–desorption processes in different environmentally relevant conditions.

The purpose of this study was to elucidate NOM influences on As sorption-desorption kinetics and speciation transformation by examining interactions of HA, as a model representative of NOM with arsenate and arsenite, in the presence of an iron-based adsorbent (IBA), as a model metal oxide in three probable conditions. Due to the importance of kinetics for the design of adsorption systems, several models have been proposed to describe adsorption kinetic data, and generally classified as adsorption reaction and adsorption diffusion models [54]. Herein we applied Lagergren pseudo first- and pseudo second-order models, which are among the most common kinetic models used by many researchers in various adsorption processes (e.g., [55,56,57]). Different environmentally relevant conditions were designed. The first scenario represented the co-presence of both HA and As species interacting with IBA. The second scenario, in which As-presorbed IBA was exposed to NOM, may also be important, as it may happen in cases, such as the initiation of passive bio-remediation of mine drainage [58,59] or the establishment of a passive remediation system to treat As-contaminated water. The third scenario was the presence of IBA preloaded with HA in an As-containing solution, which may represent the conditions when As-laden water is treated by an adsorbent system previously exposed to NOM.

## 2. Materials and Methods

### 2.1. Materials and Chemicals

Although application of iron oxides coated porous solids has been well studied for As removal (e.g., [60,61]), little information is available on diatomite, as widespread lightweight sedimentary rock, coated with iron oxide [9]. Iron oxide coated diatomite (IOCD) was thus used as a model metal-based adsorbent and prepared using a method described by Pan *et al.* [9]. The prepared IOCDs are denoted as IOCD-X_Y_, where X denotes the number of iron oxide coating times and Y denotes the particle size of the diatomite (mm). IOCD-2_0.11_ was employed in this study due to its high efficiency in As removal based on previous findings [9]. The characteristics of IOCD can be found in Pan *et al.* [9]. Na_2_HAsO_4_·7H_2_O (Alfa Aesar, Heysham, Lancashire, UK) and NaAsO_2_ (GR grade, Sigma, St. Louis, MO, USA) were used as sources of arsenate and arsenite, respectively, to prepare experimental solutions of specified concentrations. Commercial HA was obtained from Aldrich Chemical and dissolved into ultrapure water (>18.1 MX cm) at pH > 10 followed by filtering through 0.45-µm acetate cellulose membranes (Advantec, Tokyo, Japan) for preparing the HA stock solution. Control samples showed no organic carbon released when using the filter membranes. HA stock solution (1000 mg/L) was kept in a glass bottle in darkness at 4 °C. Adsorption experiments were performed within 4 weeks after the stock solution was prepared. The pH of the samples (7.5) was adjusted using 1 M HNO_3_ or NaOH, and the expectable pH value was manually maintained to the desired value until the end of the experiments. All reagents were of analytical grade, and de-ionized (DI) water was used throughout for the preparation of solutions and dilutions wherever required.

### 2.2. Batch Experiments

The kinetics of As adsorption onto IBA is an important factor in determining As retention in aquatic and soil environments. Different batch-experiment scenarios were performed to determine the nature of As speciation and adsorption-desorption kinetics, as described in the following. (A) A simultaneous reactions among HA, As(III or V) and IBA (IBA-As-HA) were performed to evaluate As adsorption behavior as well as its redox transformation; (B) HA was added to desorb As(III or V) from As-presorbed IBA ((IBA-As)+HA); and, (C) As(III or V) was adsorbed onto HA-presorbed IBA ((IBA-HA)+As) to determine the role of HA on the kinetics of the adsorption-desorption and redox states of As species in liquid phase where As enters NOM-containing soil or sediment. Batch experiments were performed using 2-L PE bottles in the dark. Samples were placed in a 360° rotator (TCLP-601P, Taiwan) with a rotation speed of 27 ± 1 rpm at room temperature for a 144 h period. The rotation speed was the same as in Pan *et al.* [9] for kinetic experiments and models, while the 144 h allow reaching the adsorption equilibrium. For scenario (A), both As species (about 5 mg/L) and HA (5 and 30 mg/L) were mixed with an IBA solution (0.2 g/L), with samples periodically taken at different time intervals for As content analysis. Note that the As concentration chosen is close to many groundwater in As-contaminated areas, like Taiwan, which is at mg/L levels. In scenario (B), arsenate and arsenite (5 mg/L) were allowed to sorb individually onto adsorbent. After equilibrium was reached, the solutions were filtered through 0.45-μm acetate cellulose membranes followed by collection of the As-presorbed IBA samples. Desorption of As by HA was tested by adding the collected IBAs to HA solution (5 and 30 mg/L). The same procedure was applied for approach (C) where the collected IBA preloaded with HA was added into the As(III or V) containing solution (5 mg/L). The suspensions were then shaken gently in darkness. Each system was allowed to re-equilibrate, with samples periodically taken at different time intervals for As content analysis. All batch experiments were conducted in at least duplicates, the average values of which are reported.

### 2.3. Analytical Methods

High performance liquid chromatography with on-line hydride generation and atomic fluorescence spectrometry (HPLC-HG-AFS) (PS Analytical, Kent, UK) was used for the measurement of As speciation in solutions. All samples were filtered before analysis and the instrument calibrated each time before use. Total As in solutions, was determined using an inductively coupled plasma–optical emission spectrometer (ICP-OES, Ultima 2000, Longjumeau, France). Each sample was injected three times, and the relative standard deviation (RSD) for the triplicate analysis was within 5%. Zeta potential of the HA in the solution was measured by a Zetasizer Model 2000 (Malvern Instruments Co., Ltd., Malvern, UK). Due to size limitation of the zeta potential analyzer, the surface charge of the IBA particles and IBA preloaded with As and HA was determined by a potentiometric titration method [62,63]. IBA (10 g/L) with a background electrolyte solution of 0.01 M KNO_3_ was first placed in the shaker for 24 h at room temperature. Titrations were then carried out using 0.1 M hydrochloride acid followed by 0.1 M sodium hydroxide, the pH values of which were measured throughout the titration process. The volume (ml) of acid or base needed to change the pH from 3 to 10 was recorded. Duplicate samples were measured with result reported as an average.

### 2.4. Kinetic Models

The Lagergren pseudo first and second order models, commonly used for sorption systems, assumed that the adsorption rate is related to the difference between the amounts of adsorbate bound at any given time *versus* the adsorbate bound at equilibrium [64,65]. The first- and second-order models can be respectively expressed as Equation (1) and Equation (2):

(1) $$q_{ads}=q_{e,ads}\left(1-e^{k_{1,ads}t}\right)$$

(2) $$q_{ads}=\frac{q_{e,ads}^{2}k_{2,ads}t}{1+q_{e,ads}k_{2,ads}t}$$

where *k_1,ads_* and *k_2,ads_* are the 1st and 2nd order adsorption rate constants (hr^−1^), in which a larger rate constant demonstrates that a shorter time is required for reaching a specific fractional uptake compared with a smaller rate constant [66], while *q_ads_* is the amount of As adsorbed at time *t* and *q_e,ads_* is the amount of As adsorbed at equilibrium.

To provide a model for desorption kinetics, which have thus far not been modeled in the literature, the present work proposes to modify the commonly used Lagergren pseudo first- and second-order kinetic models for adsorption and adapt them to desorption. The modified models consider the As desorption reaction as the rate-limiting step, with the As-sorbed sites (amounts) at the sorbent surface as the reactant and the desorption rate depending on the quantity of these As-sorbed sites (amounts).

The pseudo 1st-order desorption model assumes that the rate of desorption is proportional to the quantity of As desorbed back into the liquid phase, with the equation expressed as:

(3) $$\frac{dq_{des}}{dt}=k_{1,des}\left(q_{e,des}-q_{des}\right)$$

where *k_1,des_* is the 1st-order desorption rate constant (hr^−1^), *q_des_* (mg/g) is the remaining amount of As bound to the sorbent at any time *t*, $\frac{dq_{des}}{dt}$ is the desorption rate (mg/(g·hr)), and *q_e,des_* is the remaining amount of As bound to the sorbent at equilibrium (mg/g).

With the assumptions that at *t = 0, q_des_(0) = q_e,ads_* and at *t→∞, q_des_ (∞) = q_e,des_* , the equation can be rewritten as:

(4) $$q_{des}=q_{e,des}+\left(q_{e,ads}-q_{e,des}\right)e^{-k_{1,des}t}$$

The modified pseudo-2nd-order model for desorption assumes that the rate of desorption is proportional to the square of the amount of As desorbed back into the liquid phase, and can be expressed as:

(5) $$\frac{dq_{des}}{dt}=k_{2,des}{\left(q_{e,des}-q_{des}\right)}^{2}$$

Rearranging Equation (5) we obtain:

(6) $$q_{des}=q_{e,des}+\frac{\left(q_{e,ads}-q_{e,des}\right)}{\left(1+\left(q_{e,ads}-q_{e,des}\right)k_{2,des}t\right)}$$

where *k_2,des_* is the 2nd-order desorption rate constant (g/(mg·hr)).

We used Equations. (1) and (2) to describe the kinetics of As(III) and As(V) adsorption onto IBA, and Equations (4) and (6) for desorption from IBA.

### 2.5 Rate Limiting Mechanism

In order to gain insight into the mechanisms and rate controlling steps affecting the kinetics of adsorption, the kinetic experimental results were fitted to Weber’s intraparticle diffusion model [67]. The kinetic results were then accordingly analyzed to elucidate the diffusion mechanism, as given below:

(7) $$q_{t}=k_{id}t^{\frac{1}{2}}+C$$

(8) $$k_{id}=\frac{6q_{e}}{R}\sqrt{\frac{D}{\pi }}$$

where *q_t_* (mg/g) and *q_e_* (mg/g) are the As uptake at time *t* and at equilibrium, respectively. *C* is the intercept and *k_id_* is the intraparticle diffusion rate constant (mg/g·h^1/2^) related to the respective intraparticle diffusion coefficient (*D*) (cm^2^/s), while *R* is the particle radius (5.5 × 10^−3^ cm). Then, based on Equation (7) *k_id_* can be evaluated from the slope of the linear plot of *q_t_ versus t^1/2^*, the intercept of which reflects the boundary layer effect [68]. The larger the intercept, the greater the contribution of the surface sorption in the rate controlling step [69]. If the regression of *q_t_ versus t^1/2^* is linear and passes through the origin, then intraparticle diffusion is the sole rate-limiting step.

## 3. Results and Discussion

### 3.1. Surface Charge Analysis

**Figure 1:**
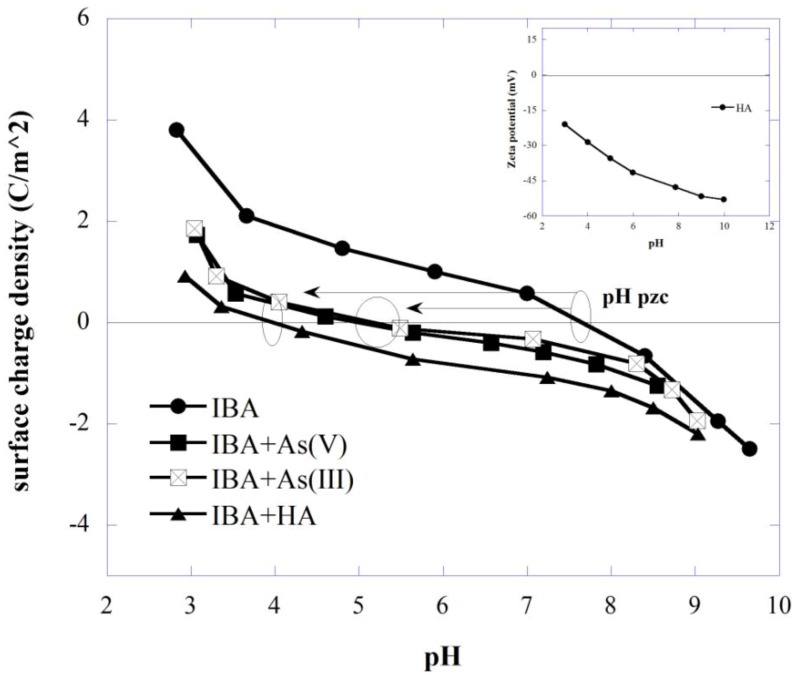
Zeta potentials of HA and surface charge density curves of bare IBA and IBA preloaded with As(III), As(V) and HA at different pH conditions.

In order to better interpret adsorption-desorption results, zeta potentials and surface charge analyses of HA and IBA were investigated to quantify the adsorbent affinity under different adsorption process conditions (Figure 1). As can be seen, HA (50 mg/L) showed negative zeta potentials in the studied pH range possibly due to the dissociation of H^+^ from the carboxylic and phenolic groups in the HA macromolecules [70]. Similar patterns were observed in other studies showing negative zeta potentials of HA at pH > 2 [70,71,72,73]. It is also apparent that HA became more negatively charged as pH value increased due to ionization of the carboxylic and phenolic functional groups [73]. The dissociation of carboxylic groups usually occurs at pH > 3, whereas the phenolic groups undergo dissociation at pH > 9 [74]. This ionization would lead to an increase of negatively charged HA molecules. Under a neutral pH condition, HA is a partially charged anion due to the deprotonation of the carboxylic groups present at the periphery of the molecules. Apparently, the carboxylic group was identified as one of the major functional group of HA [75,76], which would play an important role in the adsorption process at neutral and acidic conditions.

Surface charge analysis for the IBA-inclusive systems was performed through potentiometric titration, which measures the dependency of the “equilibrium”—pH values of the solid dispersion on the added volume of titrant (strong acid or strong base). If the amount of solid is sufficiently high and the initial amount of acid or base is known, such a titration would yield a charge (*σ_0_*) related to the adsorption and desorption reactions of protons and hydroxide ions [77]. The surface charge density was expressed in C/m² and determined by [78]:

(9) $$\sigma_{0}=\frac{(C_{A}-C_{B}+\left[OH^{-}\right]-\left[H^{+}\right])\;F}{Am}$$

where *C_A_* and *C_B_* are the respective concentrations of acid and base in mol/L needed to reach a given point on the titration curve, *[H^+^]* and *[OH^−^]* in mol/L are the concentrations of H^+^ and OH^−^ converted from pH and adjusted by the Davis equation [78], *F* is the Faraday constant (96490 C/mol), *A* is the specific surface area of employed IBA (93,000) [9] in m^2^/kg, and *m* is the concentration of IBA in kg/L.

The surface charge of bare IBA and IBA preloaded with either HA or As is plotted as a function of solution pH and shown in Figure 1. In the case of bare IBA, the surface charge decreases with increasing pH, and is negative for solution pH > pH_pzc_ (7.8) and positive for pH < pH_pzc_. The oxide surfaces charge is reported to be associated with adsorption or desorption of protons bound to the surface hydroxyl groups [79,80].

As shown in Figure 1, the surface charge of IBA shifted to more acidic pH levels in the presence of As and HA; consequently, the pH_pzc_ decreased to pH 5.1 and pH 4, after adsorption of As and HA, respectively. Whereas HA was negatively charged in the range of 3–10, in this case, the negatively charged HA adsorbed onto and neutralized the positive charges of IBA. A shift of pH_pzc_ is a very good indication that As and HA show significant adsorption affinity towards iron oxide-based surfaces which can explain the desorption of As due to HA adsorption and surface charge modification. The decrease of the surface charge after arsenic adsorption implies that adsorption occurred over the whole pH range, even when the iron oxide mineral surface charge was negative. This observation also suggests that As was bound as an inner sphere complex (specific adsorption) onto iron oxide surfaces, and so adsorption could occur at pH levels above the pH_pzc_ [79]. As can be seen, the decrease in the surface charge was not equally distributed and the change in surface charge was less significant at high pH values. Compared to HA, the decrease magnitude of the surface charge at a given pH due to As adsorption was less significant at high pH such that the surface charge values were similar at pH 9. The higher changes of surface charge as a result of HA adsorption, suggesting both inner- and outer-sphere HA complexation mechanisms, consistent with other organic matter sorptions onto different iron oxides [81,82].

### 3.2. As Adsorption in the Ternary System of As-IBA-HA

#### 3.2.1. As Removal Efficiency in Simultaneous Presence with HA

**Figure 2:**
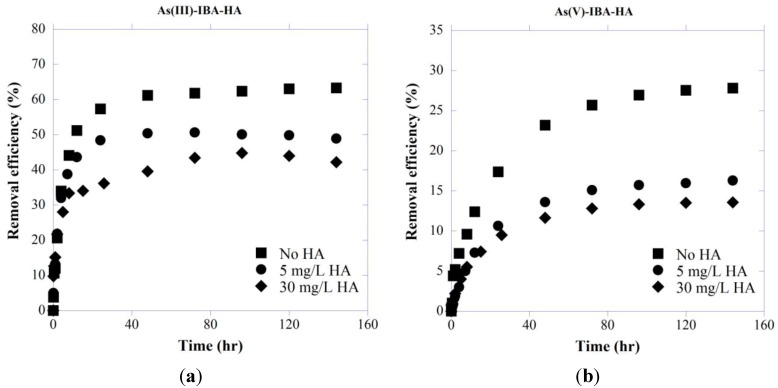
Effect of HA concentrations on removal efficiency of (**a**) As(III) and (**b**) As(V) by IBA with initial As concentration of 5 mg/L at pH 7.5.

Figure 2 presents the removal efficiency of As by IBA calculated as follows:

(10) $$RE\;\left(\text{\%}\right)=\;\frac{C_{0}-C_{t}}{C_{0}}\times 100$$

where C_0_ is the initial concentration of adsorbate, and C_e_ is the concentration of adsorbate at time t.

As(III) is known to be neutral in the pH range of 2–9, whereas As(V) is negatively charged. As As(III) uptake is less affected by solution pH, it has been shown that at neutral pH values (6 < pH < 8) As(III) is generally better adsorbed than As(V) [15]. As shown by zeta potential analysis (Figure 1), HA is negatively charged at pH > 3 and considering negative ionic form of As(V) at studied pH (7.5), increased repulsion between HA and arsenate species result in stronger competition between these two negatively charged compounds for adsorption sites which can explain less efficient removal of As(V) in presence of HA. As can be seen in Figure 2, systems without HA were more effective in As removal and different concentrations of HA significantly influence the removal efficiency of both As species.

#### 3.2.2. As Redox Transformation of System As-IBA-HA

It has been shown that NOM may catalyze both the oxidation and reduction reactions among chemical species, partly through the quinone-mediated formation of free radicals [47,83,84] where mineral (hydro)oxides may act as a surface catalyst or an electron-transfer intermediate. Redman *et al.* [47] reported the redox transformation of both As species by experimental NOM samples. Oxidation-reduction reactions involving metal (hydro)oxides and sedimentary organic matter have also been attributed to As contamination in groundwater beneath the southern Carson Desert, in Nevada [85].

**Figure 3:**
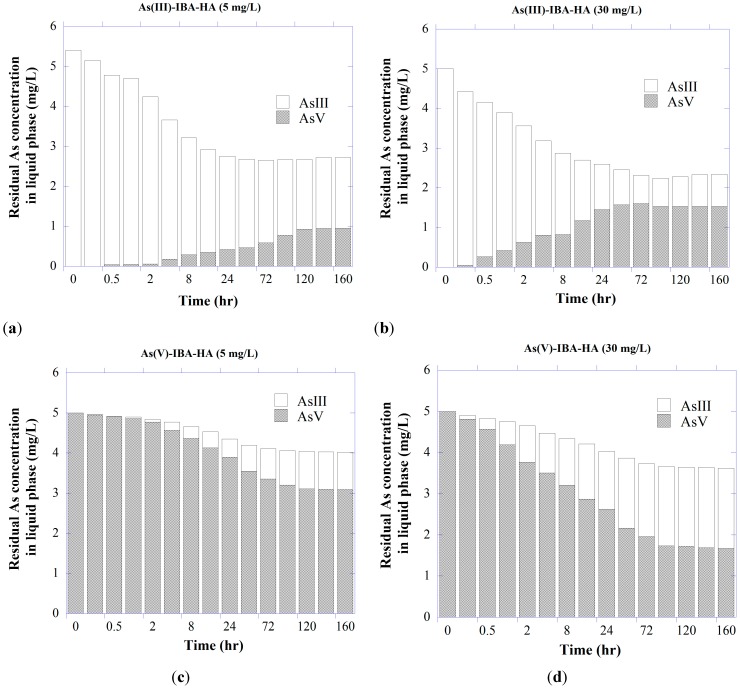
Redox transformation of (**a**, **b**) As(III) and (**c**, **d**) As(V) in liquid phase of the ternary As-IBA-HA system with initial As concentration of 5 mg/L at pH 7.5.

As shown in Figure 3, different concentrations of HA facilitated redox transformation of As species, with the result that in the system containing arsenite with 30 mg/L HA, ~63% of the free As was present as arsenate at the end of the experiment. When compared with oxidation, the reduction ability was lower, as there was ~47% of free arsenite for the system containing As(V). Overall, the results illustrate the potentially great influence of HA exposure upon As redox speciation. Possible explanations include either inherent metal contents of the HA associated with its ability to facilitate transformation of As species or HA structure itself as the redox-active agent [86]. It is also possible that solid surface may act as a catalyst, in which the IBA’s surface mediates the redox reactions between HA and As species [47].

#### 3.2.3. Kinetics of As Co-Sorption with HA onto IBA

**Figure 4:**
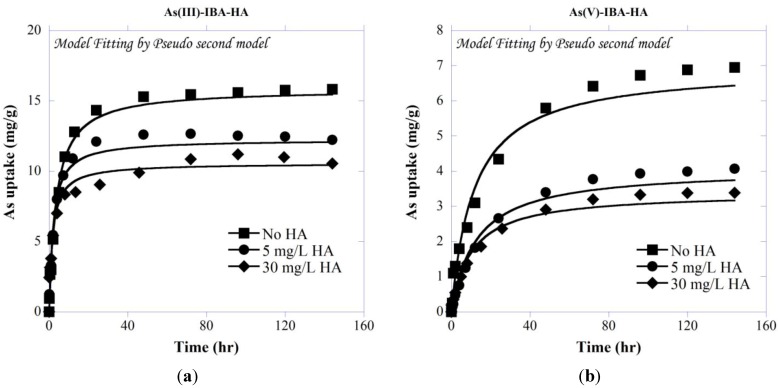
Kinetic adsorption of (**a**) As(III) and (**b**) As(V) on IBA in presence of different concentrations of HA with initial As concentration of 5 mg/L at pH 7.5.

Kinetic adsorption of total As species onto IBA in the presence of different concentrations of HA is presented in Figure 4. As shown, As uptake is clearly suppressed with increasing HA concentrations. The adsorption was fast at time < ~24 h, and approached to equilibrium after about 72 h and 96 h for As(III) and As(V), respectively. The Lagergren’s first- and second-order model parameters of all ternary systems are presented in Table 1.

As presented in Table 1 and Figure 4, the studied systems were found to follow both pseudo first-and second-order kinetic models, with slightly lower Root Mean Square Error (RMSE) for pseudo second-order model. It is noted that due to the smaller molecular weight of As species compared with HA [87], the As transport rate within the adsorbent’s pores is expected to be faster and not affected by the presence of different concentrations of HA.

It has been shown that HA and inorganic anions such as sulfate or phosphate compete with each other for sorption sites on metal oxides [88,89]. Accordingly, oxyanions such as As may also compete with HA, and adsorb onto IBA to a lesser extent. The kinetics of As adsorption is in agreement with previous studies, showing a fast initial adsorption followed by a slower phase (e.g., [9,90]).

### 3.3. Desorption of As from As-Presorbed IBA in HA Solution

#### 3.3.1. Desorption Efficiency

**Figure 5:**
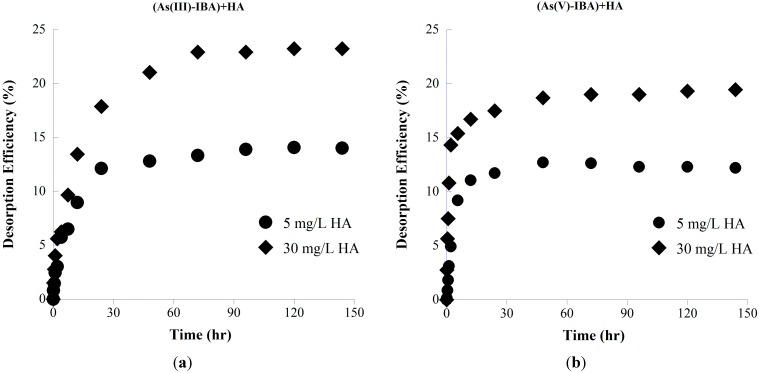
Desorption kinetics of (a) AsIII and (b) AsV using different concentrations of HA with initial As concentration of 5 mg/L at pH 7.5.

The desorption efficiencies were calculated using different concentrations of HA based on their efficiency in As detaching from the adsorbent’s surface. For each As species, and at each HA concentration, the desorption efficiency DE (%) was calculated as:

(11) $$DE=\left(\frac{q_{e,ads}-q_{e,des}}{q_{e,ads}}\right)\times 100$$

where *q_e,ads_* is the amount of As sorbed onto the adsorbents before desorption takes place, while *q_e,des_* is the amount of remaining As sorbed onto the sorbents after desorption achieved equilibrium (mg/g) (Figure 5). As shown, higher concentrations of HA (30 mg/L) produces a higher desorption efficiency for both As species compared with that for lower HA concentrations. Higher desorption efficiency of As(III) than As(V), could be due to either highly stable IBA-As(V) complexes over the surface at studied pH [91,92] or stronger repulsive forces between IBA surface and negatively charged HA as the adsorption of negatively charged species increases the number of negative charges on the surface [93,94,95].

#### 3.3.2. As Redox Transformation

**Figure 6:**
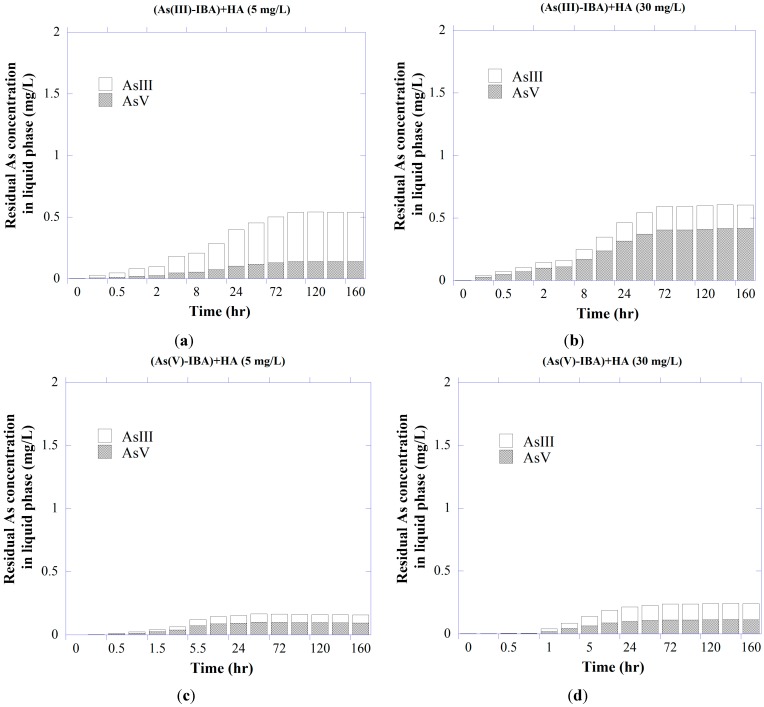
Desorption and redox transformation of (**a**,**b**) As(III) and (**c**,**d**) As(V) from As-presorbed IBA in the ternary (As-IBA)+HA system.

The influences of HA on redox speciation of desorbed As were again evident (Figure 6), showing similar patterns in the oxidation of As(III) to ternary As-IBA-HA experiments (given in Figure 2). HA showed much greater tendencies to oxidize As(III) in the presence of IBA. Possible explanations for the increased As oxidation are that the IBA acted as either a surface catalyst or an electron-transfer intermediate between oxidized HA functional groups and As(III) [47,96].

As shown in Figure 6, after a period of 96 h, HA oxidized arsenite into arsenate to a level of about 70% of the total free As in the presence of 30 mg/L HA, where arsenate reduction occurred to a lower extent (~50%). The surface of adsorbent and HA polyfunctional structure may play a significant role in the catalysis of the As(III) oxidation through an electron transfer mechanism [6].

#### 3.3.3. Kinetics of Adsorption and Desorption

**Figure 7:**
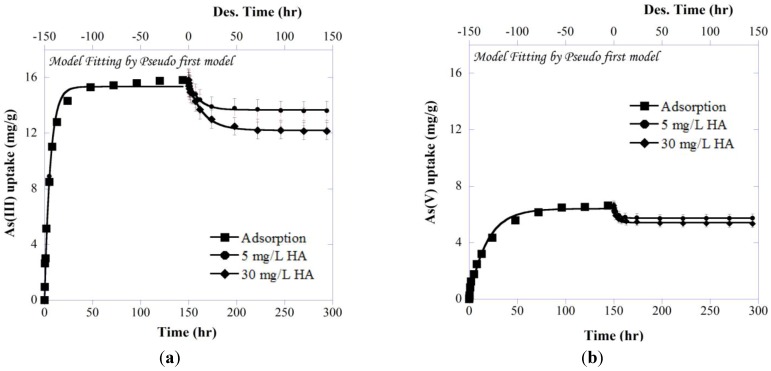
(**a**) As(III) and (**b**) As(V) adsorption–desorption curves at pH 7.5 and room temperature. The zero point on second x-axis indicates the time of HA addition. Lines in the adsorption/desorption show predictions using pseudo first order sorption kinetic model.

The initial amount of presorbed As onto IBA was about 16 ± 0.15 and 7 ± 0.18 mg/g for As(III) and As(V), respectively. Figure 7 shows kinetic results of the batch adsorption–desorption experiments and the model fits using the pseudo first-order model, as slightly better fits were found compared to the second-order model (see RMSE in Table 1). In the figure, the As uptake is plotted as a function of time, where time zero corresponds to the beginning of the As adsorption experiment. It should be noted that adsorption curves for two HA concentrations coincide due to the same initial As concentration. For desorption part, time zero corresponds to the time of HA addition, and the first data point is obtained after 10min of reaction. As can be seen, an increase in HA concentration leads to an increase in As desorption. Analyses of the aqueous phases showed that HA caused the release of significant amounts of As into solution, ranging from 15%–26% and 12%–19% of the total presorbed As(III) and As(V), respectively. Control experiments lacking HA were analyzed for total As by ICP-OES (data not shown) and showed negligible aqueous As.

**Table 1:** Model Fitting Parameters of pseudo 1st and 2nd order models for adsorption and desorption kinetics of As on IBA.

System	Adsorbate	HA Conc.		Adsorption				
				k		r^2 a^	RMSE ^b^	
			1st	2nd	1st	2nd	1st	2nd
Co-adsorption As-IBA-HA	As (III)	0	0.200	0.020	0.99	0.99	0.10	0.09
		5	0.201	0.023	0.98	0.99	0.18	0.15
		30	0.212	0.026	0.95	0.98	0.30	0.17
	As (V)	0	0.049	0.010	0.99	0.98	0.13	0.10
		5	0.050	0.011	0.98	0.99	0.19	0.14
		30	0.051	0.013	0.98	0.99	0.21	0.13
HA-preloaded (HA-IBA)+As	As (III)	0	0.190	0.020	0.99	0.99		
		5	0.069	0.009	0.98	0.98	0.17	0.25
		30	0.046	0.006	0.97	0.98	0.27	0.26
	As (V)	0	0.048	0.010	0.99	0.99		
		5	0.020	0.008	0.98	0.98	0.16	0.19
		30	0.015	0.006	0.99	0.98	0.11	0.15
**System**	**Adsobate**	**HA Conc.**		**Desorption**				
				**k**		**r**	**RMSE**	
**As-Preloaded**			**1st**	**2nd**	**1st**	**2nd**	**1st**	**2nd**
(As-IBA)+HA	As(III)	5	0.08	0.07	0.99	0.99	0.07	0.09
		30	0.07	0.05	0.99	0.99	0.11	0.18
	As(V)	5	0.06	0.8	0.99	0.93	0.01	0.12
		30	0.34	1.2	0.98	0.94	0.07	0.14

a. coefficient of determination; b. Root Mean Square Error.

Sorbed arsenite (Figure 7a) was released to a greater extent than was for sorbed arsenate (Figure 7b) under different concentrations of HA, which is consistent with the generally greater mobility of As(III) in aqueous systems. The HA ability to mobilize sorbed As, over time scales of at most 48 h in length, was the most striking result of this experiment. Although the extent of As desorption varied, each concentration of HA desorbed a significant amount of As compared to the controls (without HA), indicating that HA may play a significant role in As fate and mobility in natural systems as well.

### 3.4. Adsorption of As onto HA-Presorbed IBA

In natural environments, humic-coated iron-oxide often exists, and interferes with the adsorption behavior of iron-oxide colloids and contaminants [97]. In these experiments, HA was presorbed onto IBA, followed by the introduction of arsenate or arsenite.

#### 3.4.1. As Removal Efficiency

**Figure 8:**
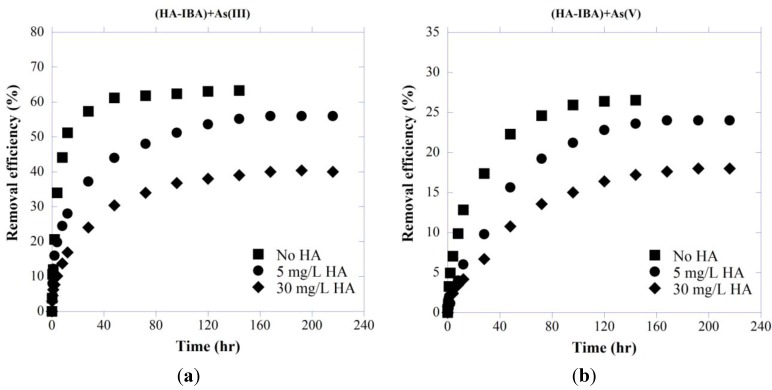
Effect of HA-presorbed IBA on removal efficiency (**a**) As(III) and (**b**) As(V) with initial As concentration of 5 mg/L at pH 7.5.

Removal efficiency of As in the system with HA-presorbed IBA is shown in Figure 8. As can be seen, IBA preloaded with HA significantly affected arsenic removal efficiency, and when compared with bare IBA, the As(III) and As(V) removal efficiency was respectively reduced about 36% and 27% in the presence of IBA presorbed with 30 mg/L HA. In contrast with neutral As(III), the inorganic As(V) species are negatively charged at the studied pH (7.5). Besides, at every pH, there is a large number of positive, neutral, and negative sites on the adsorbent’s surface. The *σ_0_* value gives the net charge density where at the zero point of charge (zpc), the number of positive sites equals the negative ones and the net charge is zero [98].

Although IBA preloaded with HA is net negative at pH 7.5, its small positive domains may be accessible, and contribute electrostatic attractions. It is thus assumed that adsorption in the studied pH region may still controlled by the minority of positive surface sites, even though the net surface charge is negative [99,100].

The partial positive surface charge of the iron oxide is thus expected to have stronger ionic bonding with As(V) than As(III) due to the electrostatic attraction [9] and this may explain the less decrease in removal efficiency of As(V) compared with As(III).

#### 3.4.2. As Redox Transformation

**Figure 9:**
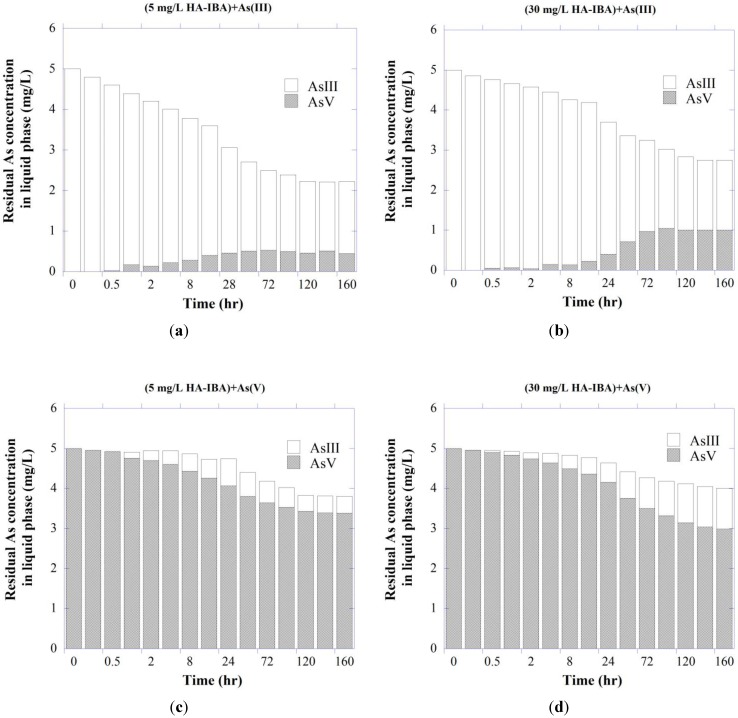
Redox transformation of (**a,b**) arsenite and (**c,d**) arsenate sorption onto HA-presorbed IBA for 168 h reaction. The initial concentration of As was 5 mg/L at pH 7.5 and symbols represent duplicate analysis.

Free As in the liquid phase again showed speciation changes in the presence of HA (Figure 9). Redox transformation for both As species occurred to a generally lower extent than that observed for As-presorbed IBA in HA solution (Figure 6), as speciation changes were respectively reduced by about 50% for both As(III) and As(V) species in HA-presorbed IBA in As solution compared with ((As-IBA)+HA) system. Perhaps, due to the sorbed HA, the IBA’s ability to mediate redox reactions between HA and As species was decreased. Clearly, the oxidation of As(III) by HA was a much more favorable process than the reduction of As(V) in all scenarios tested, as expected in these oxygenated systems. This implies that HA interference with arsenite sorption may have been tempered by its tendency to oxidize the arsenite to a form that may sorb more stably to natural materials [47].

#### 3.4.3. Kinetics of As Adsorption onto IBA Preloaded with HA

**Figure 10:**
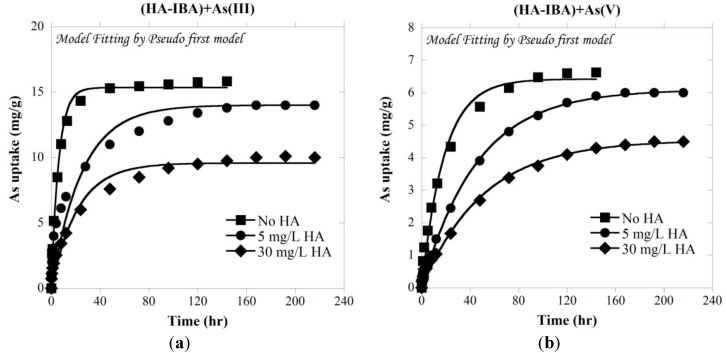
Kinetic adsorption of (**a**) As(III) and (**b**) As(V) on IBA preloaded with different concentrations of HA and initial As concentration of 5 mg/L at pH 7.5.

The adsorption equilibrium of As onto HA-presorbed IBA displayed relatively slow kinetics and lower adsorption capacity when compared to that onto bare IBA. As shown in Figure 10, presence of HA decreased the ability of As to sorb onto the IBA, causing about 14%–50% and 5%–15% more As(III) and As(V) to remain in solution with 5 and 30 mg/L HA compared with the As and IBA only system.

Table 1 shows that the obtained *k_1,ads_* and *k_2,ads_* rate constants decreased with increasing HA concentrations in the system of IBA-preloaded with HA. It can be concluded that in a sorption system with a lower HA concentration faster kinetics can be expected. The HA-presorbed IBA showed lower rate constants of adsorption for both As(III) and As(V) compared with the rate constants for the adsorption systems of arsenic only and arsenic plus HA. This observation may be attributed to two reasons. One is that the presorbed macromolecular HA may occupy some of the adsorbent surface sites, and the other is that the HA may block some of the available pathways for As transport into the adsorbent’s pores, which results in reducing the accessibility of As species for adsorption sites. Due to the partial site coverage and pore blockage, the As adsorption capacity of HA-presorbed IBA is also lower than that of As on IBA (with no HA); however for lower concentration of HA, the total As uptake of HA-preloaded IBA shows higher capacity than the system with As and HA simultaneously (As-HA-IBA). It is expected that at lower HA concentrations, only limited amount of HA is expected to adsorb onto IBA, leading to less site coverage and pore blockage on IBA. Although more studies are needed to confirm mechanisms and quantify the adsorption extents, complexation of As with HA may be involved in the increase of As adsorption with HA-presorbed IBA [76]. Complexation of As with the pre-adsorbed HA may lead to an increase of adsorption capacity of As. In addition, in the system of As and HA simultaneous adsorption, the available HA mass in the solution is much higher than that in the HA-presorbed system. Complexation of As with the dissolved HA may also reduce the available As to adsorb on IBA.

On the other hand, the organic ligands of previously adsorbed HA sequester surface sites and may block the diffusion of As to the oxide surface [70]. Additionally, the limited number of adsorption sites, results in a pronounced decrease of the overall amount of adsorption [101]. Furthermore, HA may competitively occupy the surface sites against As species [102].

Importantly, HA inhibited the adsorption of introduced arsenite even more effectively than that of arsenate (Figure 8 and Figure 10), which is consistent with the generally greater mobility of As(III) compared to As(V) in natural aquatic environments [103]. Compared with bare IBA, about 36% and 27% less uptake were observed for As(III) and As(V), respectively in 30 mg/L HA. Moreover, as stated earlier, in contrast to As(III), the inorganic As(V) species, H_2_AsO_4_^−^ and HAsO_4_^2−^, are negatively charged at the studied pH here (7.5), with stronger bonding with IBA through electrostatic attraction of the partial positive surface charge of the iron oxide and negatively charged As(V) [9]. Davis and Bhatnagar [104] showed that the sequential order of adsorption of dissolved and adsorbed HA considerably changed the adsorption characteristics of cadmium onto hematite where for 10^−6^–10^−4^ M Cd(II), 5–10 mg/L HA, and 4 h reaction period, Cd(II) adsorption capacity was found in the following order: Cd(II) adsorbed before HA > simultaneous adsorption of Cd(II) and HA ≈ HA adsorbed before Cd(II).

### 3.5. Sorption Mechanism

**Figure 11:**
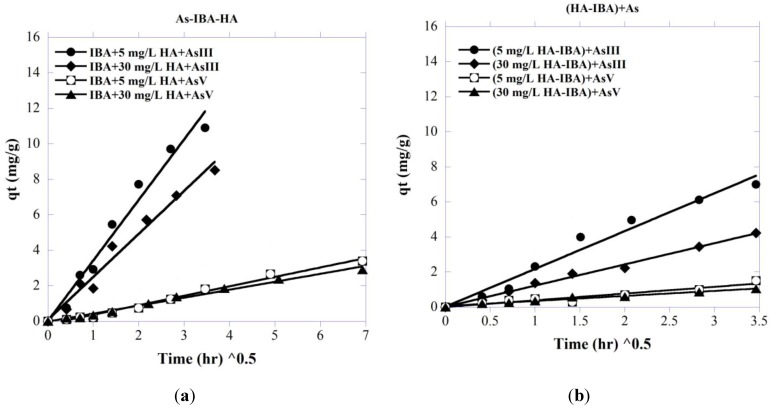
Weber and Morris intra-particle diffusion plots for removal of As species (5 mg/L) in (**a**) co-adsorption with HA and (**b**) IBA preloaded with HA at different HA concentrations, pH = 7.5 and room temperature.

Figure 11 represents plots of *q_t_ versus* $\sqrt{t}$ (Equation (7)) for the adsorption of As(III or V) with the co-presence of HA onto bare IBA as well as HA-presorbed IBA, to obtain Weber-Morris kinetic parameters. In the system containing both As and HA simultaneously, and with an As initial concentration of 5 mg/L and HA = 5 and 30 mg/L, the data points represent linear plots for the experimental data. The Weber-Morris kinetic parameters, along with the correlation coefficients for both adsorbate–adsorbent systems, are given in Table 2. As shown, a linear relation of q_t_ and t^0.5^ is demonstrated and the coefficient for the intraparticle diffusion model is equal to one, where the small value of the intercept could be negligible [105]. The general linear patterns of q_t_ and t^0.5^ shown in the figures may be attributed to the instantaneous utilization of the most readily available adsorbing sites on the internal surface of the adsorbent, indicating that intraparticle diffusion of As species onto IBA is the main rate-controlling step for the systems studied [106], including the system with co-presence of As and HA in solution and the system with sorption sites occupied by HA.

The values of *k_id_* for HA preloaded systems indicate that the intraparticle diffusion rate for As species is higher at lower concentrations of HA. As pore dimensions decrease due to the adsorption of the competing HA, the free path for molecules to travel within the transport pores becomes smaller (with possible pore blockage occurring), and hence the transport rate decreases.

From Equation (8), it can be seen that *k_id_* is proportional to both *q_e_* and *D^1/2^*. The obtained results of the systems with co-sorption of As and HA (Table 2) showed that HA did not significantly influence the diffusivity (*D*) of As into IBA; this is probably due to the fact that HA molecules are much larger than As [52], and so the diffusion coefficient of HA molecules is expected to be smaller and the diffusional transport of As in the intraparticular pores faster and not affected by the presence of HA. Other studies have also revealed that the presence of organic ligands had no significant effect on the diffusion coefficients of trace elements (e.g., [107,108]); hence the *k_id_* should be proportional to *q_e_*. As also confirmed by the pseudo second-order adsorption kinetic model (Figure 4), the equilibrium concentration *q_e_* decreases accordingly with the increasing HA concentrations. Consequently, the constant *k_id_* decreases with increasing HA concentration due to the decreasing *q_e_*. For the systems with HA-preloaded IBA, as presented in Table 2, both *q_e_* and *D* decrease with increasing HA concentrations, which is possibly due to blockage of certain transport pathways in IBA, leading to smaller pore diffusion coefficients. Consequently, the constant *k_id_* decreases with increasing HA concentration may be attributed to the decreasing *q_e_* and *D^1/2^*.

**Table 2:** Weber-Morris kinetic parameters of As sorption onto IBA in the presence of HA.

System	HAConc. (mg/L)	k_id_ (mg/(g h))	q_e_ (mg/g)	D (cm^2^/s)	r^2^
Co-adsorption of HA and As					
(As(III)-IBA-HA)	5	3.40	12.29	3.9×10^−7^	0.98
	30	2.42	9.97	3.8×10^−7^	0.98
(As(V)-IBA-HA)	5	0.53	4.01	4.4×10^−8^	0.99
	30	0.44	3.20	4.5×10^−8^	0.99
HA Pre-loaded					
(HA-IBA)+As(III)	5	2.15	14.00	8.4×10^−8^	0.98
	30	1.20	9.57	7.0×10^−8^	0.99
(HA-IBA)+As(V)	5	0.38	6.00	2.5×10^−8^	0.99
	30	0.28	4.54	1.4×10^−8^	0.99
With No HA					
(IBA-As(III))	0	3.98	15.34	3.9×10^−7^	0.99
(IBA-As(V))	0	0.60	6.71	4.6×10^−8^	0.98

## 4. Conclusion

Arsenic adsorption-desorption kinetics and aqueous speciation were evaluated in three types of equilibrated ternary systems consisting of As, HA and IBA. The first system was composed of the co-presence of both As and HA interacting with IBA (As-HA-IBA). The second system comprised pre-equilibrated As–IBA with subsequent addition of HA ((As-IBA)+HA), where As was allowed to sorb individually onto the adsorbent and after it reached equilibrium, As-presorbed IBA samples were collected and introduced into HA solution. The third consisted of the pre-equilibrated HA–IBA with the subsequent addition of As ((HA-IBA)+As), where similar with the second system, the collected HA-presorbed IBA samples were added into an As-containing solution. Adsorption kinetic data were fitted well using the Lagergren pseudo-first and second-order adsorption kinetic models. To provide a model for desorption kinetics, the commonly used kinetic models for adsorption were modified and adapted to desorption, where the remaining amount of As bound to the surface was considered as the rate-determining concentration. While As uptake was clearly suppressed with increasing HA concentration in the system with the co-presence of HA and As, different concentrations of HA caused the release of significant amounts of previously sorbed As into solution. As exposure to IBA preloaded with HA, may weaken the ability of IBA for As uptake and slightly slow the adsorption kinetics. Based on the model fit with the Weber-Morris model, intraparticle diffusion of As species onto IBA was found to be the main rate-controlling step. The experiments conducted for redox speciation of As revealed that presence of dissolved and sorbed HA may facilitate the redox transformation of As species, with higher oxidation ability than reduction in all scenarios tested. The findings of this study showed that HA has a great potential for influencing sorption behavior and redox transformation of As species through interacting with iron oxide surfaces and/or with As itself, and thus may play a significant role in the fate, transfer and release of As from terrestrial environments into the groundwater.
